# Myelin Oligodendrocyte Glycoprotein-Associated Disorders Post-ChAdOx1 Vaccination

**DOI:** 10.7759/cureus.23197

**Published:** 2022-03-15

**Authors:** Boby Varkey Maramattom

**Affiliations:** 1 Neurology, Aster Medcity, Kochi, IND

**Keywords:** myelin oligodendrocyte glycoprotein-associated disorders, post-chadox1 vaccination mogad, post-vaccination mogad, chadox1 vaccination, mogad

## Abstract

ChAdOx1 nCoV-19 vaccine (AstraZeneca) has been associated with rare adverse events following vaccination such as thrombosis with thrombocytopenia syndrome, inflammatory myositis, and autoimmune encephalitis. Para-infectious or post-infectious myelin oligodendrocyte glycoprotein-associated disorders (MOGAD) have been reported in association with coronavirus disease. However, post-vaccine MOGAD (PV-MOGAD) has not yet been reported. Here, we report three cases of PV-MOGAD who presented with a prolonged severe headache after the ChAdOx1 vaccination. Other features of MOGAD such as optic neuritis or tumefactive demyelination appeared much later. Aseptic meningitis can be a presenting feature of PV-MOGAD. When patients present with a severe headache after the ChAdOx1 vaccination, PV-MOGAD should be considered along with thrombosis with thrombocytopenia syndrome.

## Introduction

The ChAdOx1 nCoV-19 vaccine is associated with very rare adverse events following immunization (AEFI), such as thrombosis with thrombocytopenia syndrome, inflammatory myositis, and autoimmune encephalitis [1-3]. Myelin oligodendrocyte glycoprotein-associated disorders (MOGAD) is an inflammatory demyelinating disorder associated with immunoglobulin G (IgG) serum MOG antibodies. MOGAD presents with optic neuritis, transverse myelitis, acute disseminated encephalomyelitis, aseptic meningoencephalitis, or pseudotumor cerebri-like presentations [4].

MOGAD has been reported with coronavirus disease 2019 (COVID-19) as a para- or post-infection [5-8]. Severe acute respiratory syndrome coronavirus 2 (SARS-CoV-2) infection is thought to elicit a host autoimmune response that precipitates MOGAD. However, post-vaccine MOGAD (PV-MOGAD) after COVID-19 vaccination has not yet been described. Here, we describe three cases of PV-MOGAD after the ChAdOx1 vaccination.

## Case presentation

Case 1

A 43-year-old woman received her first dose of the ChAdOx1 vaccination. Six days later, she started developing severe bifrontal headaches and photophobia. On admission, 15 days later, she was noted to have terminal neck stiffness and grade I papilledema. Cerebrospinal fluid (CSF) examination was normal. Antinuclear antibody (ANA) profile and routine workup were negative. Magnetic resonance imaging (MRI) of the brain showed scattered areas of sulcal and leptomeningeal enhancement in the frontotemporal areas. Her serum COVID-19 anti-spike antibody was strongly positive. She was treated with steroids (intravenous (IV) dexamethasone 4 mg BD for seven days) and antibiotics (ceftriaxone) with a presumptive diagnosis of aseptic meningitis. When her headaches improved after a week she was discharged. One week later, she presented with a worsening headache. This time MRI of the brain showed a right temporal heterogeneously enhancing lesion with mass effect for which underwent a right temporal craniotomy and excision. Bacterial and fungal cultures did not show any growth. Histopathology showed cores of neuroparenchyma with dense interstitial and perivascular infiltrates of macrophages and foamy histiocytes admixed with lymphocytes.

Myelin (Luxol fast blue) stains highlighted demyelinated areas with macrophages displaying cytoplasmic myelin debris. The features were consistent with tumefactive demyelination. At this point, serum was highly positive for MOG IgG antibody by indirect immunofluorescence on transfected cells.

Two weeks later, her headache recurred and she was started on IV methylprednisolone 1 g/day for three days and two doses of rituximab 1 g IV one month apart. She remained asymptomatic at the two-month follow-up. A repeat serum MOG IgG was moderately positive.

Case 2

A 26-year-old woman developed a severe headache 10 days after her first dose of the ChAdOx1 vaccination (Visual Analog Scale (VAS) score, 8/10). She was treated symptomatically and referred to neurology after two months when she developed limb paraesthesia and sequentially decreased vision in both eyes over two weeks. MRI of the brain showed bilateral optic neuritis. CSF examination showed 25 cells, all lymphocytes with normal protein and sugar, and oligoclonal bands. MOG IgG antibody was strongly positive. She was started on IV methylprednisolone 1 g/day for five days, followed by oral prednisolone 40 mg and mycophenolate 1 g/day. Her headaches disappeared, and steroids were tapered five months later. Two weeks after steroids were stopped, she presented with severe headaches (VAS, 9/10) and vomiting. MRI of the brain and spine were normal; however, a repeat CSF study showed 85 cells with 58% lymphocytes, normal protein, and glucose.

A CSF meningoencephalitis panel, neuronal antibody panel, ANA, and antineutrophil cytoplasmic antibodies were negative. MOG IgG antibody was again strongly positive. She was started on IVIg 2 g/kg over five days and IV methylprednisolone 1 g/kg for five days. As she had persistent severe headaches for two weeks, she was administered rituximab 1 g. Her headache disappeared by week four. She was discharged on oral prednisolone 40 mg/day and mycophenolate 1 g/day. Rituximab 1 g was readministered at one month. A final diagnosis of recurrent PV-MOGAD meningitis was made.

Case 3

**Figure 1:**
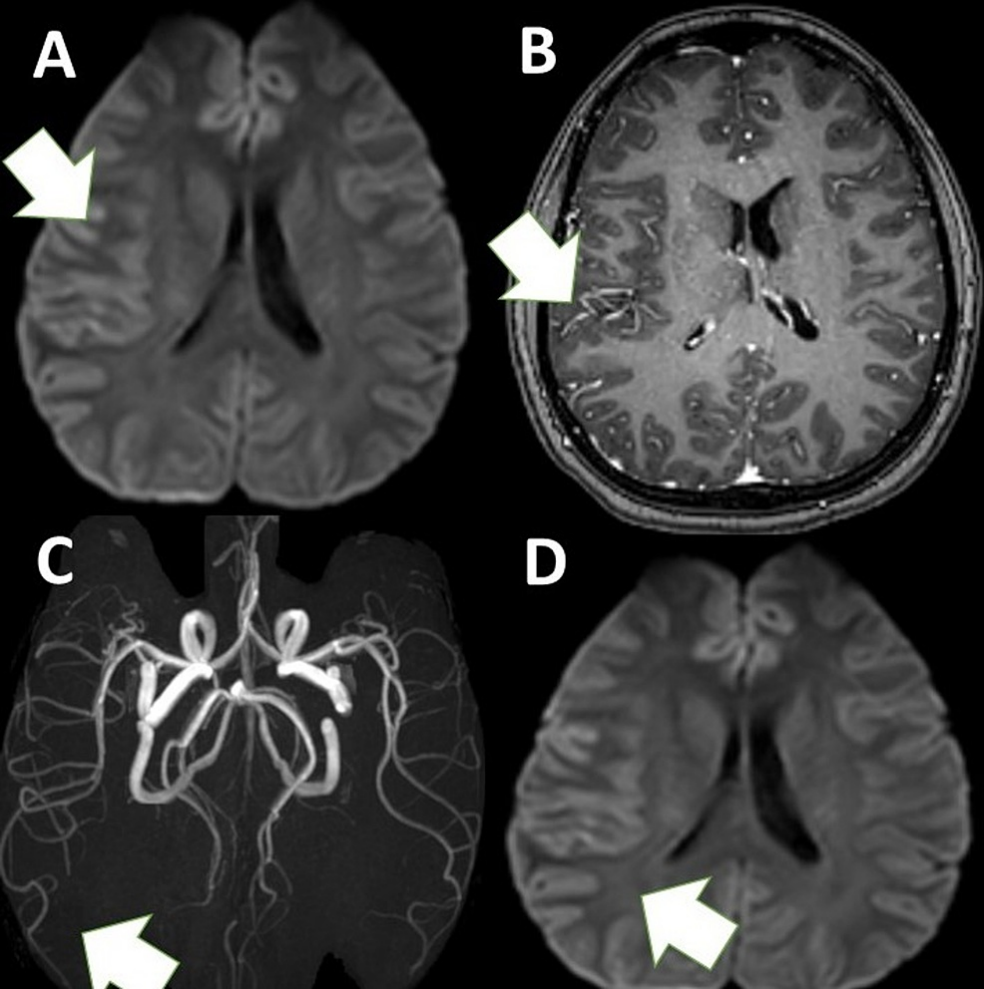
MRI images. (A) Right-sided FLAMES. (B) MRA showing right hemispheric hypervascularity (white arrow). (C) Contrast-enhanced T1-weighted axial image showing focal leptomeningeal enhancement. (white arrow). (D) Diffusion-weighted MRI image showing right hemispheric T2 shine through (no corresponding ADC changes). FLAMES: FLAIR-hyperintense lesions in anti-MOG associated encephalitis with seizures; FLAIR: fluid-attenuated inversion recovery; MRA: magnetic resonance angiography; MRI: magnetic resonance imaging; ADC: apparent diffusion coefficient

A 20-year-old woman developed severe right-sided headache, right ear pain, and neck pain the day after her second dose of the ChAdOx1 vaccination. On day three, she presented with fever, right hemiparesis, dysarthria, and right-sided numbness. She was discharged when an outside MRI of the brain with magnetic resonance angiography was normal. Five days later, she was admitted to our hospital when she developed a right-sided severe headache (VAS, 7/10) and transient left hemiparesis. Repeat MRI of the brain with contrast showed right hemispheric fluid-attenuated inversion recovery MRI hyperintense cortical lesions with overlying leptomeningeal enhancement (Figure 1).

CSF MOG antibodies were positive. CSF showed 30 cells (all lymphocytes) with normal glucose and protein levels. She was treated with IV methylprednisolone 1 g/day over five days. Rituximab 1 g IV was added when repeated hemiplegic migraine occurred. Table 1 presents a summary of the clinical details of the three patients.

**Table 1:** Clinical details of our patients. CSF: cerebrospinal fluid; MRI: magnetic resonance imaging; COVID-19: coronavirus disease 2019; IV: intravenous; ANA: antinuclear antibody; ANCA: antineutrophil cytoplasmic antibodies; MOG: myelin oligodendrocyte glycoprotein; Ig: immunoglobulin; FLAMES: FLAIR-hyperintense lesions in anti-MOG associated encephalitis with seizures; FLAIR: fluid-attenuated inversion recovery

Case	Clinical features	Treatment offered
Case 1	Symptom onset six days after the first dose. Recurrent headaches, terminal neck stiffness, and grade I papilledema. CSF was initially normal. MRI initially displayed leptomeningeal enhancement, followed by tumefactive demyelination. Serum COVID-19 anti-spike antibody was strongly positive. Brain biopsy proved demyelination	IV dexamethasone 4 mg BD for seven days, IV methylprednisolone 1 g/day for three days, two doses of IV rituximab 1 g. Improvement was noted at 60 days
Case 2	Symptom onset 10 days after the first dose. Headaches, followed by optic neuritis. ANA and ANCA negative. CSF examination showed 25 cells, all lymphocytes with normal protein and sugar, and oligoclonal bands. CSF meningoencephalitis panel, neuronal antibody panel, ANA, and ANCA were negative. Serum MOG IgG antibody was repeatedly positive	IV methylprednisolone 1 g/day for five days, followed by oral prednisolone 40 mg, and mycophenolate 1 g/day. IVIg 2 g/kg over five days and IV methylprednisolone 1 g/kg for five days. Two doses of rituximab 1 g IV, followed by oral prednisolone 40 mg/day and mycophenolate 1 g/day. Recurrent headaches. Improvement noted by month seven
Case 3	Severe right-sided headache and right ear pain One day after the second dose of the ChAdOx1 vaccination. Alternating hemiplegia was noted. CSF meningoencephalitis panel, neuronal antibody panel, ANA, and ANCA were negative. Serum MOG IgG antibody was repeatedly positive. CSF showed 30 cells (all lymphocytes) with normal glucose and protein levels Repeat MRI showed FLAMES	IV methylprednisolone 1 g/day over five days. Rituximab 1 g IV for two doses. Improvement noted in three weeks

## Discussion

Our cases fulfilled diagnostic criteria for MOGAD and were diagnosed with PV-MOGAD. The initial presentation with isolated headaches and the tardy development of a demyelinating disorder (30-60 days later), alternating hemiparesis, and late MRI changes delayed the diagnosis of MOGAD [9]. Similar to other autoimmune ChAdOx1-AEFI (which present within six weeks after vaccination), our patients developed symptoms 10 days after vaccination. The subsequent disease evolution, recurrence, relapse on steroid discontinuation (case 2), and MOG antibody persistence for months suggest the triggering of a MOGAD requiring long-term therapy. Case 2 had a refractory prolonged headache that took over a month to resolve, despite multiple pain medications and immunomodulators. Case 3 had headaches, alternating hemiparesis, and unilateral cortical encephalitis compatible with FLAMES (FLAIR-hyperintense lesions in anti-MOG-associated encephalitis with seizures). As PV-MOGAD developed after adenovector vaccination, we hypothesize that the host response to components of the ChAdOx1 vaccine induced anti-MOG antibodies.

As of December 23, 2021, more than 1.39 billion COVID-19 vaccines have been administered in India. Of these, >90% have received the ChAdOx1 vaccine (1.2 billion). In the state of Kerala, over 25 million people have been vaccinated against COVID-19. In India, the crude annual incidence of CAE is approximately 8.35-10 per million [10].

## Conclusions

The worldwide incidence of post-vaccination encephalitis is approximately 0.4-0.8 per million ChAdOX1 vaccinees and 0.2 per million with the mRNA vaccine (BNT162b2). In our population, we estimated the crude incidence of PV-MOGAD at 0.08 per million (3 per 25 million). This is an order of magnitude less than other PVE cases and two orders of magnitude less than CAE. This confirms the safety of the COVID-19 vaccination. However, post-ChAdOx1 vaccine headache should also arouse the suspicion of an evolving MOGAD.
